# Assessment of age-related change of the ocular support system

**DOI:** 10.3389/fbioe.2023.1146828

**Published:** 2023-07-10

**Authors:** Ahmed Makarem, Ahmed Abass, Fangjun Bao, Ahmed Elsheikh

**Affiliations:** ^1^ School of Engineering, University of Liverpool, Liverpool, United Kingdom; ^2^ Faculty of Engineering, Port Said University, Port Fouad, Egypt; ^3^ School of Optometry and Ophthalmology and Eye Hospital, Wenzhou Medical University, Wenzhou, Zhejiang, China; ^4^ Beijing Advanced Innovation Center for Biomedical Engineering, Beihang University, Beijing, China; ^5^ National Institute for Health Research (NIHR), Biomedical Research Centre at Moorfields Eye Hospital NHS Foundation Trust, UCL Institute of Ophthalmology, London, United Kingdom

**Keywords:** eye, orbit, cornea, sclera, whole eye moevement

## Abstract

To estimate the material stiffness of the orbital soft tissue in human orbits using an inverse numerical analysis approach, which could be used in future studies to understand the behaviour under dynamic, non-contact tonometry or simulate various ophthalmological conditions. Clinical data were obtained for the left eye of 185 Chinese participants subjected to a complete ophthalmic examination, including tests by the Corvis ST and Pentacam. 185 numerical models of the eye globes were built with idealised geometry of the sclera while considering the corneal tomography measured by the Pentacam. The models were extended to include representations of the orbital soft tissue (OST), which were given idealised geometry. The movement of the whole eye in response to an air-puff directed at the central cornea was examined and used in an inverse analysis process to estimate the biomechanical stiffness parameters of the OST. The results indicated a weak correlation of *E*_*t*_ with the progression of age, regardless of the stress at which *E*_*t*_ was calculated. However, there was evidence of significant differences in *E*_*t*_ between some of the age groups. There was statistical evidence of significant differences between *E*_*t*_ in the age range 20< *years* < 43 relative to *E*_*t*_ in OST with age ranges 43< *years* < 63 (*p* = 0.022) and 63< *years* < 91 (*p* = 0.011). In contrast, *E*_*t*_ in OST with age ranges 43< *years* < 63 and 63< *years* < 91 were not significantly different (*p* = 0.863). The optimised mechanical properties of the OST were found to be almost four times stiffer than properties of fatty tissue of previous experimental work. This study consolidated previous findings of the role of extraocular muscles on the ocular suppor system. In addition, the rotation of the globe during corvis loading is suggested to be of posterior components of the globe and shall be further investigated.

## 1 Introduction

Eye orbits are bony sockets contained within the skull in which the eyes and the orbital soft tissue (OST) are situated (Bron, 1997). Besides its function to hold the contents of the ocular system, the orientation of the orbit affects the visual field of mammals (Heesy, 2004). The confined space of the bony orbit is mainly filled with the OST, which acts as a shock mitigator in case of sudden or traumatic impacts to the eye or head (Liu et al., 2013). This function and the need to allow the eye globe to move freely upon the contraction and relaxation of the extraocular muscles (EOM) means that the adipose fatty tissue within OST must possess a relatively low stiffness (Katch et al., 1991; Heesy, 2004; Comley and Fleck, 2012). This low stiffness causes distinctive posterior movement of the eye globe and rotation in the nasal direction when subjected to air-puff tonometry (Boszczyk et al., 2017), and these movements inevitably affect the measurement accuracy of the intraocular pressure (IOP).

The structure of the OST plays a major role in enabling the tissue to act as a shock absorber while allowing the unhindered movement of the intraorbital structures in their respected degrees of freedom (Schoemaker et al., 2006). The OST is composed of EOM, optic nerve, adipose fatty tissue (AFT), and other fibrous connective tissues (Hwang et al., 2019). Bremond et al. identified two parts of the AFT; the outer, extra-conical fat tissue and the central, intra-conical fat (Bremond-Gignac et al., 2004), with this distinction being a consequence of the cone formed by the extra-ocular muscles along with the organisation of the conjunctival tissue. Mesoscopic and histological differences were identified between the two parts with the outer and inner parts constituting thick and thin conjunctival septa, respectively. These differences were thought to be related to the mechanical role of the two parts where the first part acted as a periorbita cushion that enabled globe rotation, while the latter had a major contribution to maintaining the position of the globe, while allowing the movement of the optic nerve in the orbit (Turvey and Golden, 2012; Rootman et al., 2022).

For quite some time, numerical modelling of the OST did not get much attention. Despite the essential findings achieved by previous ocular modelling studies (Stitzel, 2002; Elsheikh et al., 2013; Aboulatta et al., 2021), the effect of the OST was ignored, and the ocular globe was provided with boundary conditions that were assumed to represent the restraints provided by the OST. These boundary conditions commonly restrained the eye’s equatorial zone against the movement in the longitudinal direction while permitting it to expand or move laterally (Elsheikh et al., 2013). In doing so, the distributed support provided by the OST was not represented and instead the support was assumed to be concentrated at the equator, leading to an unphysiological concentration of stress at this location. The representation of the OST support in this form also ignored the soft support offered against dynamic loadings of the ocular globe, which could cause inaccuracies in simulating the globe’s response. The recent technological advances within numerical solvers allowed a significant increase in computational power, which enabled further studies and more representative, and complex, modelling of OST support (Al-Sukhun et al., 2006; Schoemaker et al., 2006; Schutte et al., 2006; Chen and Weiland, 2011; Boszczyk et al., 2017; Jannesari et al., 2018; Guo et al., 2019; Hwang et al., 2019). These studies included research to evaluate the orbital fat supporting role offered to the globe during its rotation—and represented the effect of shear between the globe and orbital fat (Schoemaker et al., 2006; Schutte et al., 2006). Yet some of these studies included assumptions of high stiffness for the globe and linear behaviour for the orbital fat, possibly affecting the study outcomes (Schutte et al., 2006). The current study builds on these earlier efforts and attempts to offer more representative hyperelastic globe and OST tissue behaviour.

An earlier study (Jannesari et al., 2018) has suggested and demonstrated that the clinical whole eye movement (WEM) and the inverse finite element analysis approach quantify the corneal and fatty tissue biomechanical parameters. The study assumed that the eye is fully supported by the adipose tissue, which is inaccurate (Lockwood, 1885; Lemke and Lucarelli, 2012; Hwang et al., 2019; Banks et al., 2020). Additionally, the cornea and the boundary conditions were oversimplified and assumed to have a symmetrical geometry; (Maloney and Iii, 1993; Kahn and Shaw, 2008); hence a 2D numerical model was used. Therefore, the authors of the current study are confident that it is vital to implicate the geometrical irregularity of the OST to represent the support offered against the dynamic loading applied to the ocular globe within the analyses (Leszczynska et al., 2018) demonstrated that the WEM during tonometry is a quantifiable parameter that could be used to assess tissue behaviour in the orbital space. Thus, within this study, 3D finite element inverse analyses were carried out for 185 ophthalmologically healthy subjects to estimate their OST’s material stiffness and investigate the age-related change within the clinical sample. The authors believe this study will widen our understanding of stress-strain behaviour and material stiffness of the orbital soft tissue under dynamic, non-contact tonometry or simulate various ophthalmological conditions. In addition, a comparison of the estimated material stiffness with the experimental will highlight the role of the extraocular muscles in the ocular support system.

## 2 Methods

The study used whole eye movement data recorded for 185 subjects under the dynamic air-puff pressure applied by the Corvis ST tonometer. A numerical model was built for each eye included in the study, simulating its tomography, axial length and IOP, and considering the eye’s tissue stiffness as indicated by the Stress-Strain Index (SSI) readings (Eliasy et al., 2019). The eye models were embedded within a medium of orbital soft tissue, whose geometrical boundary surface was provided by Beijing Advanced Innovation Centre for Biomedical Engineering (BAICBE) at Beihang University based on skull MRI scans of a healthy 27-year-old Asian female (Geng et al., 2018).

### 2.1 Clinical data

A fully anonymised database of 185 Chinese ophthalmologically healthy subjects was retrospectively reviewed, see Table 1. According to the University of Liverpool research ethics policy, approval for this record review using fully anonymised secondary data was ruled unnecessary. Nonetheless, written informed consent was obtained from each participant to use their data in research. The study was conducted according to the tenets of the Declaration of Helsinki as set out in 1964 and revised in 2013.

**TABLE 1:** Age groups used in the current study.

Age group	Age range	Number of samples	Mean age±SD
	(Years)		(Years)
Young	20–43	100	29.8 ± 5.4
Middle-aged	> 43–63	50	51.2 ± 6.7
Old	> 63-91	35	72.9 ± 6.0

Earlier studies suggest that orbital health conditions, such as thyroid orbitopathy, affect WEM in response to the air pulse produced by Corvis. (Leszczynska et al., 2018; Hwang et al., 2019). Therefore, all participants were subject to a complete ophthalmic examination including tests using the Corvis and Pentacam (OCULUS Optikgeräte GmbH; Wetzlar, Germany). Subjects with a history of use of hypotonic therapies, glaucoma, previous eye disease or ocular surgery were excluded. For consistency, one clinician carried out Corvis examinations for all participants. All exams were individually reviewed by an experienced corneal specialist to ensure that only good-quality scans were included in the study.

**FIGURE 1:**
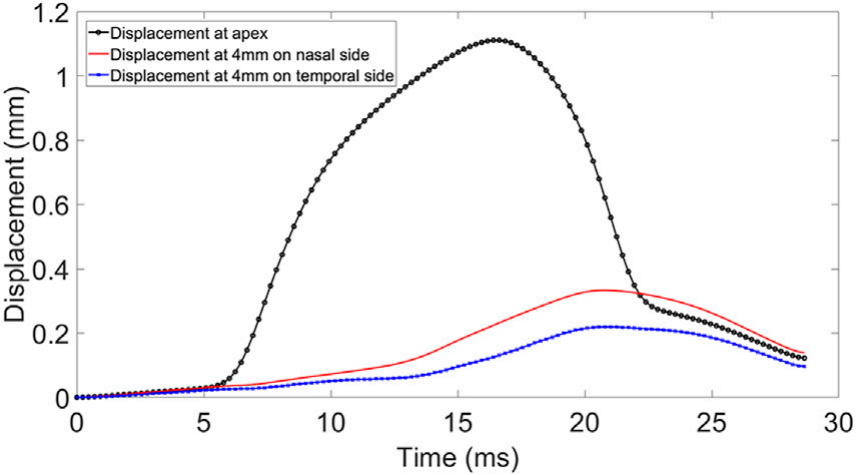
Displacement at apex, 4 mm nasal and temporal side of the cornea during Corvis ST procedure.

The Corvis ST provided a cross-sectional view along the central horizontal meridian of the cornea every 0.23 ms during the air puff application. These cross-sections covered the corneal apex and a 4 mm distance on each of the temporal and nasal sides. Analysis of the cross-sectional views led to the deformation profile at the apex and the 4 mm temporal and nasal points. While the apical deformation included both corneal deflection and the WEM, see Figure 1. the deformations at the temporal and nasal points were mainly caused by the WEM but also included some posterior deformation between these points and the eye’s posterior pole (Vinciguerra et al., 2016).

### 2.2 Finite element modelling

#### 2.2.1 Model generation

**FIGURE 2:**
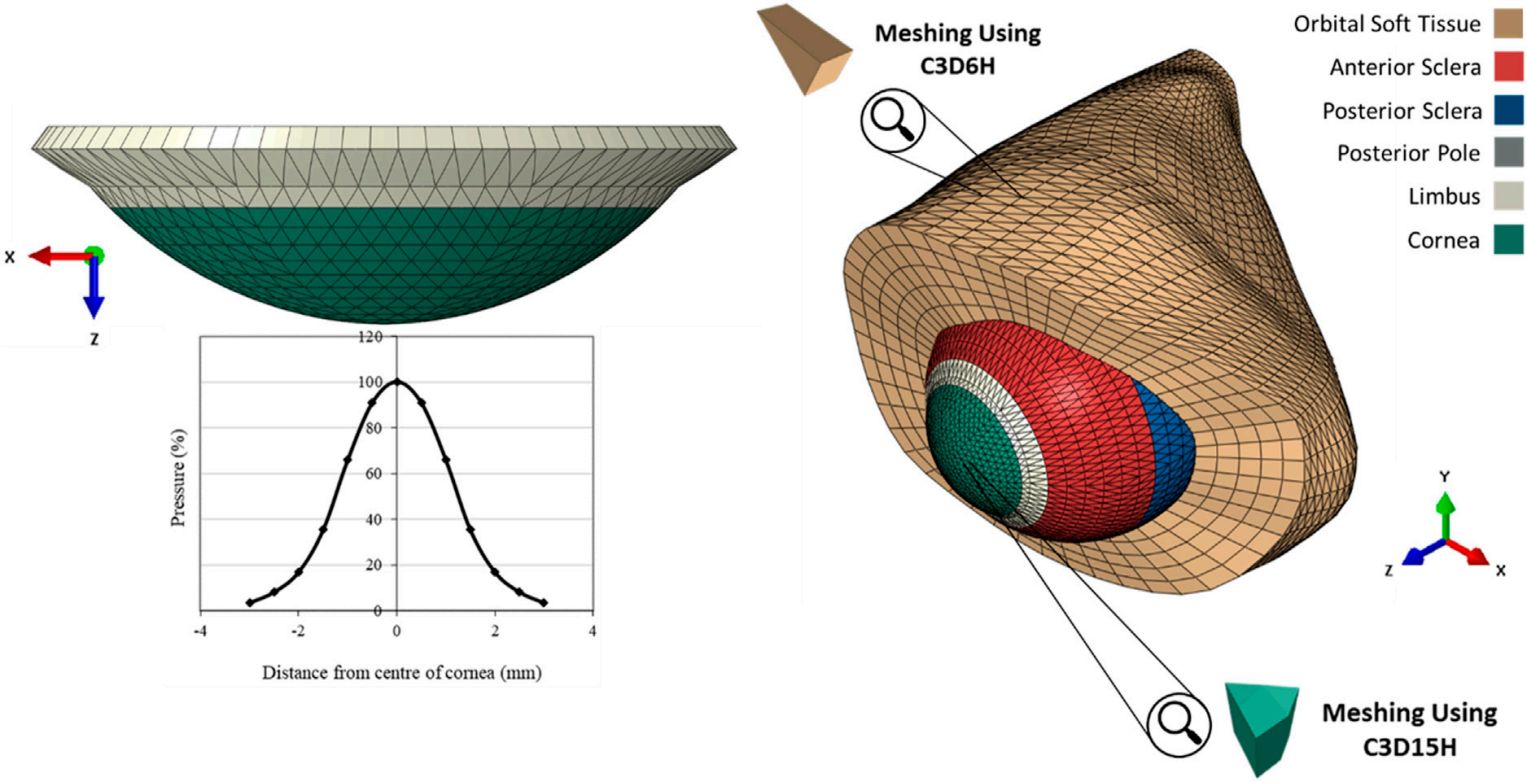
The pressure distribution on the cornea during an air puff test, and the Globe-Orbit numerical model that was used for the inverse analysis process.

The finite element models (FEM) of the eye were constructed using a custom-built MATLAB code described in an earlier study, (Whitford et al., 2015), while a further code was developed herein to build models of the OST. The models were built for analysis by the finite element analysis (FEA) package Abaqus (Abaqus/CAE 6.13-3, Dassault Systèmes Simulia Corp.). Models of the eye globe included 23232 fifteen-noded elements (C3D15H) organised in 1 layer with 16 element rings in the cornea and 72 rings in the sclera. On the other hand, the OST models included 35640 six-noded elements (C3D6H) organised in 5 layers as shown in Figure 2.

The eye models adopted several common attributes reported in earlier studies including the asphericity of both anterior and posterior surfaces of the cornea. The models adopted the corneal profile recorded for each eye by the Corvis ST at no external pressure and assumed rotational symmetry of corneal topography. The models also assumed a spherical shape for the scleral external surface with a radius of 11.5 mm (Whitford et al., 2016). It adopted a variable thickness that changed linearly from 1.2 times the peripheral corneal thickness (PCT) at the posterior pole, to 0.8 times PCT at the equator and a peripheral corneal thickness (which was greater than CCT by 150 *μ*m) at the limbus (Elsheikh et al., 2013). The models also assumed the corneal stroma had a weak inter-lamellar adhesion (Elsheikh, 2010).

A technique was developed to mesh irregular unsymmetrical shapes, such as the orbital cavity, while ensuring that most elements maintained a consistent shape and size throughout. In this technique, equally spaced points on the longitudinal axis of the combined model were selected, from which radial lines with equal angular spacing were ejected to coincide with the orbital cavity surface. The radial lines were then divided equally to locate intermediate points on each line. These intermediate points, in addition to the orbital surface points and points on the longitudinal axis, formed the nodes between which the elements were formed. This process was used for the whole length of the optic nerve except that within the depth of the eye globe, the radial lines extended from the external surface of the eye model to corresponding points on the orbital surface.

Darcy et al. (2008) carried out a Magnetic Resonance Imaging (MRI) characterisation of orbital changes with age. The outcome of that study suggested a significant increase in the anterior inferior periocular soft-tissue volumes, mainly due to the expansion of fatty tissue in this region. It was also suggested that this trend might be the reason for the lower eyelid prominence, affecting the eye globe’s anterior-posterior position within the orbital space. Other studies Fledelius and Stubgaard (1986); Kaye et al. (1992); Pessa et al. (1999); Knudtzon (1949); Lang et al. (1985); Ahmadi et al. (2007), showed changes in exophalmetery value with progression of age, and the most recent study Ahmadi et al. (2007) showed an average reduction of 0.066 *mm*/*year* in ocular protrusion within both genders. In that study, ocular protrusion was measured from the farthest lateral part of the orbital rim to the corneal apex.

**FIGURE 3:**
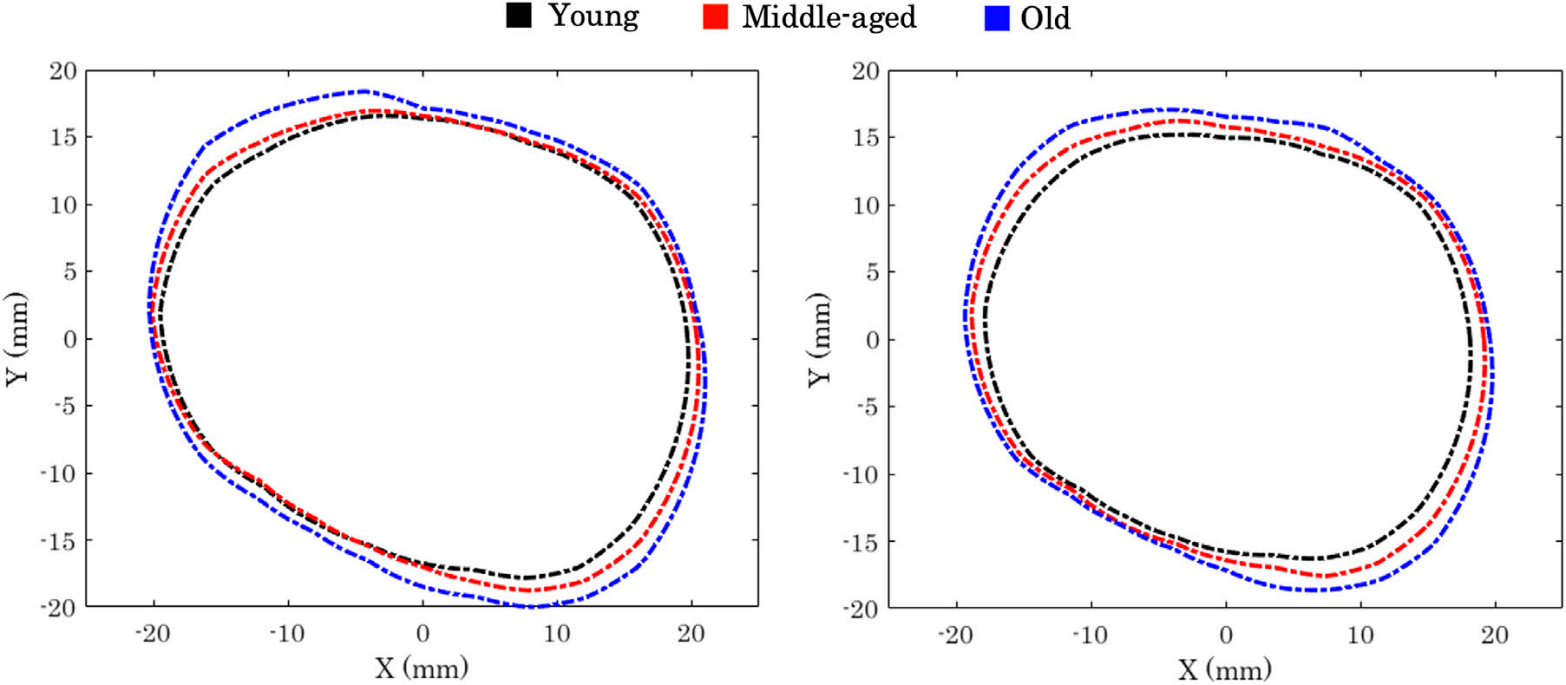
Orbital aperture age-related variation in males (left) and females (right) Kahn and Shaw (2008).

Kahn and Shaw (2008) conducted a three-dimensional computed tomographic study to outline the effect of age on the orbital aperture. The study used a 3D reconstruction of CT scans, followed by measuring orbital aperture width as the distance between the *frontozygomatic suture* and the posterior *lacrimal crest*. This study demonstrated significant changes between age and gender groups in orbital aperture width and area. They also reported that the area increase in the aperture was not uniform across the boundary. In Caucasian males, most of the increase in area was due to a receding boundary at the superior-nasal portion of the rim and a recession of the entire inferior orbital rim, Figure 3.

#### 2.2.2 Corvis pressure distribution

**FIGURE 4:**
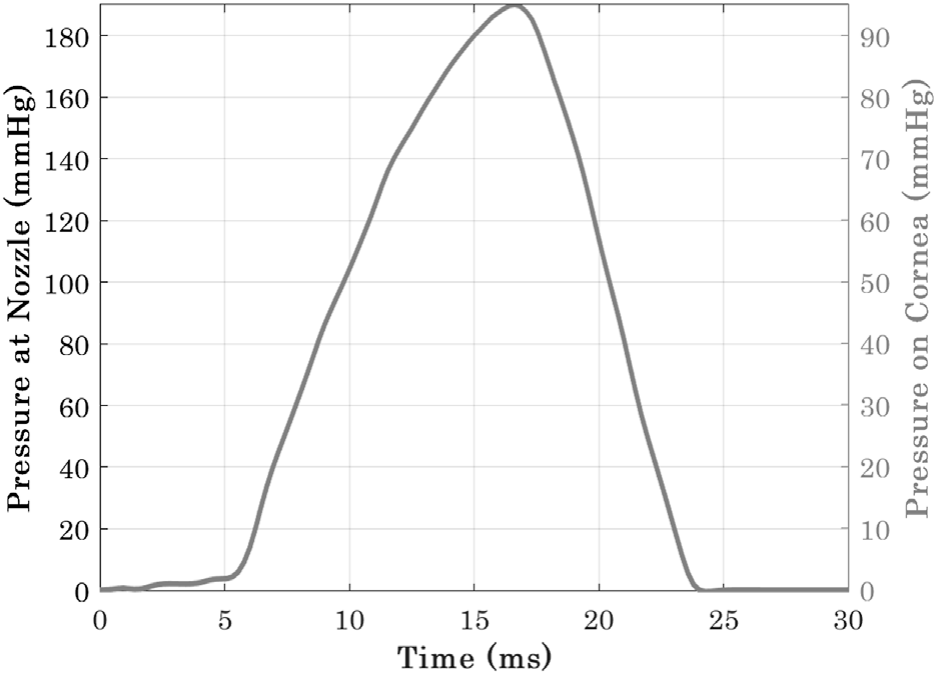
Pressure-time distribution at Corvis nozzle and the cornea Eliasy et al. (2019).

Corvis applies a jet of pressurised air to the central region of the cornea for a duration of 30 *ms*. A prior experimental study Eliasy et al. (2019) has concluded that air-puff fired from the nozzle has twice the pressure magnitude than the one in contact with the corneal surface, see Figure 4. It was necessary to use an accurate time-pressure distribution. Therefore, 130 pressure profiles of healthy clinical subjects were assessed intervalley throughout the air-puff procedure. It was established that all pressure profiles follow the same trend, where the standard deviation was below 4.3% of maximum applied pressure at the nozzle; hence, only one pressure-time distribution was used in the remainder of the project. In addition, an earlier study obtained the pressure distribution applied on the corneal apex and the spatial reduction in pressure away from the apex and towards the limbus. Joda et al. (2016), see Figure 2. Henceforth, all numerical simulations adopted the mean clinical time-pressure distribution and the spatial-pressure distribution.

#### 2.2.3 Material models

Previous efforts Elsheikh et al. (2009); Geraghty et al. (2012) took place to experimentally quantify stress-strain behaviour of various regions of the globe −the cornea and sclera. Another study Elsheikh et al. (2010) has followed with performing an optimisation analysis to obtain constitutive Ogden material parameters, which provided similar material behaviour for all global regions. In a similar matter, another study (Comley and Fleck, 2012) has carried out an optimisation process to obtain Ogden constitutive material model parameters for the AFT. Therefore, in this study the globe’s fifteen-noded and the OST’s six-noded numerical models, relied on Ogden Constitutive material model. Abaqus Theory Guide Documentation has provided hyperelastic Ogden strain energy equation, see Eq. 1.

$$U=\overset{N}{\underset{n=1}{\sum }}\frac{2\mu_{i}}{\alpha_{i}}\left({{\bar{\lambda }}_{1}}^{\alpha_{i}}+{{\bar{\lambda }}_{2}}^{\alpha_{i}}+{{\bar{\lambda }}_{3}}^{\alpha_{i}}-3\right)+\overset{N}{\underset{n=1}{\sum }}\frac{1}{D}{\left(J_{el}-1\right)}^{2i}$$ (1)

Where *U* is strain energy per unit volume, *μ* the shear modulus, *α* the strength hardening exponent, *N* the function order, $\bar{\lambda_{i}}$ principal stretch in each of the Cartesian planes, *D* is compressibility parameter and *J*_*el*_ is particle volume. All ocular tissue regions were assumed almost in-compressible with Poisson’s ratio of 0.48 Bao et al. (2017); Yu et al. (2013). A number of previous studies have used Ogden material model and proved its ability to represent ocular tissue material behaviour Eliasy et al. (2019); Kotecha (2007); Ogden (1997); Moran et al. (2014). Therefore, this material model required no further investigation during this study and age-related Ogden material parameters were used, Table 2.

**TABLE 2:** Controlling parameters of Ogden constitutive material model in relation to age as obtained from experimental data Elsheikh et al. (2010).

Age (Years)	*μ*(*MPa*)				*α*			
	Cornea	Anterior sclera	Equatorial sclera	Posterior sclera	Cornea	Anterior sclera	Equatorial sclera	Posterior sclera
0	0.104	1.678	0.922	0.433	119.8	31.543	41.521	53.016
25	0.115	1.913	1.081	0.554	119.8	35.303	43.876	53.016
50	0.132	2.224	1.291	0.743	119.8	40.265	46.983	53.016
75	0.157	2.633	1.568	1.096	119.8	46.815	51.084	53.016
100	0.197	3.174	1.934	1.830	119.8	55.458	56.494	53.016

#### 2.2.4 Convergence study

A convergence study was carried out in two steps to determine the optimum mesh density of the numerical models used in this study. The first step concentrated on the cornea of the eye globe and involved 12 model representations with the number of elements ranging between 9,408 and 110592. In all 12 models, the OST was represented by the same mesh with 35640 elements, and the focus was on the displacement of the corneal apex under external air pressure. The analysis results of the 12 models allowed the selection of an optimum mesh density in the eye models, which was used in the remainder of the study. The second step then concentrated on the optimum mesh for the OST model. In this work, the optimum eye mesh determined in the first step was adopted, while the OST model had a number of elements ranging between 10692 and 128700 in 12 new models. The displacement of the corneal apex in all models was again used in selecting the optimum density of the OST mesh.

#### 2.2.5 Inverse analysis

A custom-built MATLAB code was constructed to generate a 15 × 5 grid of *μ* (5 × 10^−5^ to 5 × 10^−3^) and *α* (0.1–50) values producing 75 combinations of the OST materials parameters. A total of 75 numerical simulations were conducted using each of the *α* and *μ* combinations. However, it was found that the root mean square error (RMSE) changed very slightly (below 1%) with changes in *α*, therefore this parameter was set at a value of 21 in all clinical cases. Within the code, the patient Corvis examination file was read to extract the deformation caused by the air puff test. Analysis of the examination files showed that beyond a 4 mm distance from the corneal apex, there was nearly no corneal deformation (less than 3% of apical deformation). Therefore, it was assumed that the displacement beyond this point was due to the whole eye movement (WEM) inside the orbit space, which was presumed due to the mechanical properties of the OST (Jannesari et al., 2018). Subsequently, two anterior corneal nodes at approximately 4 mm radius on either side (temporal and nasal) of the apex were monitored. In order to eliminate the effect of eye rotation that was observed in both clinical data and numerical predictions (Boszczyk et al., 2017), the mean of the temporal and nasal displacements was considered when assessing the match between the WEM obtained clinically and numerically. The mismatch between the Corvis eye movement results and the models’ predictions was calculated for the 15 models using the following objective function:

$$RMSE=\sqrt{\frac{\sum_{n=1}^{N}{\left(WEM_{\mathrm{NUMERICAL}}-WEM_{\mathrm{CLINICAL}}\right)}^{2}}{N}}$$ (2)

where RMSE is the root mean square of mismatch between the whole eye movement measured clinically (*WEM*_*CLINICAL*_) and predicted numerically (*WEM*_*NUMERICAL*_). N is the number of time steps of the simulation (N = 72). Once all 15 simulations were completed, the *μ* − RMSE results were fitted to a 2nd order polynomial with a local RMSE minimum, from which the µvalues that could give the minimum RMSE were determined.

#### 2.2.6 Statistical analysis

Statistical analyses were carried out using IBM SPSS Statistics 24, IBM, Armonk, New York, U.S. Data were expressed as mean, standard deviation and range. Pearson correlation analysis was performed to study the relationship of the tangent modulus (*E*_*t*_) in different age groups Table 1 at three stress levels (0.15 × 10^−3^, 0.30 × 10^−3^, 0.45 × 10^−3^ MPa). These stress levels covered most of the stress range, to which the OST was subjected in the Corvis ST numerical simulations. In these analyses, *p* values smaller than 0.05 were indicative of statistical significance.

## 3 Results

### 3.1 Convergence study

The eye globe models were analysed under IOP and Corvis ST air pressure with meshes that ranged in the number of elements from 9,408 to 110592. The mesh density study showed a 7.94% change in apical displacement when the number of elements increased from 9,408 to 23232, while a much smaller change in displacement of 0.01% occurred with a further increase in the number of elements from 23232 to 110592 elements. As a result, eye models with 23232 elements were used in the remainder of this study. Models including both the globe and the orbit were also analysed with mesh densities involving numbers of elements between 10692 and 128700 (while fixing the number of elements representing the globe at 23232). The mesh density study showed a 4.34% change in the corneal apical displacement with an increase in the number of elements from 23760 to 35640. A much smaller change in displacement of 1.06% resulted in a further increase from 35640 to 105750 elements. Consequently, a mesh density with 35640 elements, arranged in 13 layers.

### 3.2 Inverse analysis

Using the “minimum least-squared error” method to determine values of the OST material parameters *μ* and *α* that provided the best possible match with the whole eye movement measured clinically resulted in RMSE between 12 and 28 *μm* (mean ± SD = 24 ± 5) for the 185 eyes considered.

**FIGURE 5:**
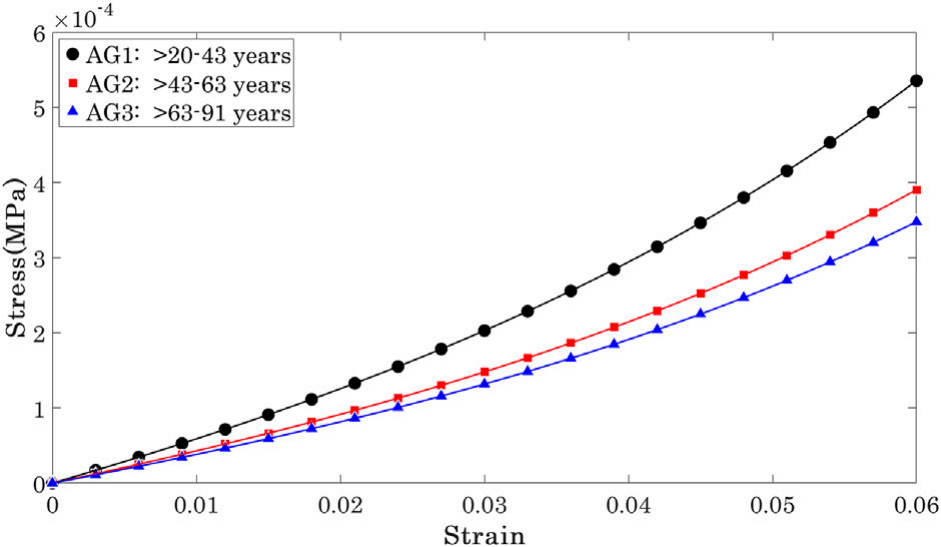
Mean Stress-strain behaviour of the OST included in the three age groups.

With the *μ* and *α* parameters determined, it was possible to plot and compare the stress-strain (*σ*-*ϵ*) relationships for individual cornea, Figure 5. To enable these comparisons, the 185 results were split equally across 3 age groups according to ages between 20 and 40 years (mean 29.83 ± 5.4, group AG1, 100 eyes), between 41 and 65 years (mean 51.18 ± 6.7, group AG2, 50 eyes) and between 66 and 91 years (mean 72.91 ± 6.0, group AG2, 35 eyes). The results showed nonlinear behaviour in all 3 groups, with AG3 exhibiting significantly lower stress values at the same strains than AG1 and AG2, while these two later groups demonstrated similar behaviour.

*E*_*t*_ was then determined as the first derivative of stress with respect to strain (*E*_*t*_ = *∂σ*/*∂ɛ*) Girard et al. (2009); Sigal et al. (2011). *E*_*t*_ showed a significant but weak negative correlation with progression of age at three stress levels (0.15 × 10^−3^, 0.30 × 10^−3^, 0.45 × 10^−3^ MPa) that covered most of the stress range to which the OST was subjected in the Corvis ST numerical procedure, Figure 5. At these stress levels, R values remained above 0.20 while *p* values were ¡0.05 between AG1 and AG2, and AG1 and AG3, although the *p*-value between AG2 and AG3 was ¿0.05.

**FIGURE 6:**
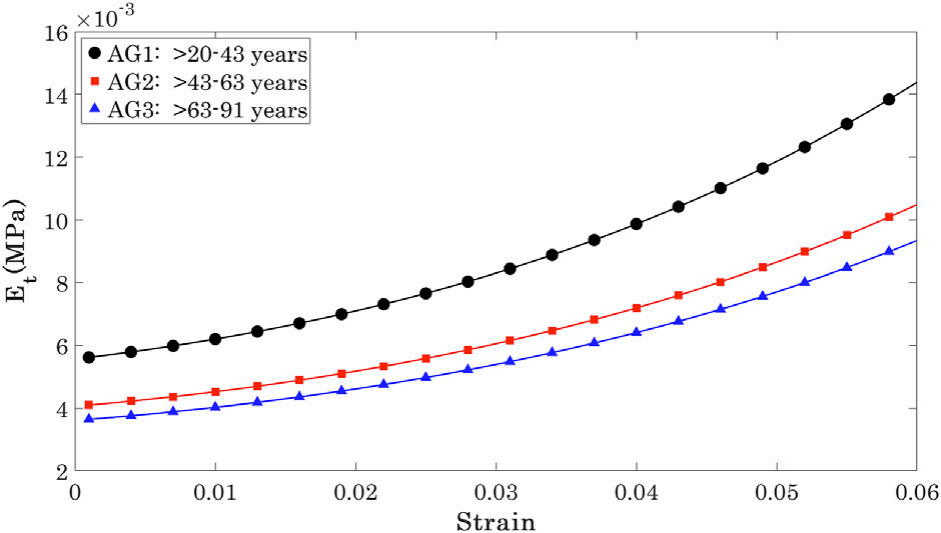
Age-related change in the tangent modulus (*E*_*t*_) calculated for all age groups.

The Et values at the three stress levels considered are plotted against age in Figure 6. The results show that while there were large differences between Et at different stress levels, the variation of Et across the age range was slight and limited to −0.27 kPa per decade at *σ* = 0.15 kPa, 0.29 kPa per decade at *σ* = 0.3 kPa, and −0.29 kPa per decade at *σ* = 0.45 kPa.

The results indicated a weak correlation of *E*_*t*_ with the progression of age, regardless of the stress at which *E*_*t*_ was calculated. However, there was evidence of significant differences in *E*_*t*_ between some of the age groups. There was statistical evidence of significant differences between *E*_*t*_ in the age range 20< *years* < 43 relative to *E*_*t*_ in OST with age ranges 43< *years* < 63 (*p* = 0.022) and 63< *years* < 91 (*p* = 0.011). In contrast, *E*_*t*_ in OST with age ranges 43< *years* < 63 and 63< *years* < 91 were not significantly different (*p* = 0.863).

## 4 Discussion

In this study, 185 numerical models were built while considering corneal tomography in each eye as measured by the Pentacam. The models also included an idealised geometry of the sclera and OST. They were analysed to simulate the performance under intraocular pressure (IOP) and air pressure experienced in Corvis tests. The models then used the corneal deformation profiles recorded by the Corvis under air pressure in an inverse analysis exercise to estimate the material behaviour of the OST. Each subject’s model was run 15 times within the inverse analysis with varying material stiffness. Material stiffness producing the most optimum deformation to its corresponding clinical data was saved for this subject along with its age and IOP, ready for further age-related analysis.

Results of the inverse analysis enabled estimation of OST’s tangent modulus (*E*_*t*_) for each of the 185 eyes included in this study. The results indicated a weak correlation of *E*_*t*_ with the progression of age, regardless of the stress at which *E*_*t*_ was calculated. However, there was evidence of significant differences in *E*_*t*_ between some of the age groups. There was statistical evidence of significant differences between *E*_*t*_ in the age range 20< *years* < 43 relative to *E*_*t*_ in OST with age ranges 43< *years* < 63 (*p* = 0.022) and 63< *years* < 91 (*p* = 0.011). In contrast, *E*_*t*_ in OST with age ranges 43< *years* < 63 and 63< *years* < 91 were not significantly different (*p* = 0.863).

Despite efforts to create age-specific numerical models of the eye globe and OST, where some geometrical (and other age-related) variables changed, material optimisation produced 1.6 ± 1 kPa as the mean optimum material stiffness for OST. Prior studiesComley and Fleck (2012); Comley and Fleck (2010) have developed a micro-mechanical model for the soft biological tissue. This proposed model suggested material stiffness of AFT being 0.4 kPa, which is significantly low (4 times softer) compared to the optimised material stiffness estimated in the current study. Hwang et al. (2019) stated that EOMs play a significant role in supporting the eye globe. Nevertheless, the discrepancy between the experimental stiffness of AFT Comley and Fleck (2012) and the optimised stiffness of the current has indirectly quantified the support provided to the eye globe through EOMs and other connective tissues such as Lockwood’s ligament. This quantification of mechanical stiffness confirms Hwang’s findings Hwang et al. (2019) and provides a comparative scenario of how various OSTs collectively support the globe against an exterior form of frontal loading.

In another study, Jannesari et al. (2018) attempted to estimate biomechanical properties of AFT using a very similar methodology to the current study; by employing an inverse analysis optimisation along with Corvis corneal deformation. However, their numerical set-up involved an idealised two-dimensional axisymmetric geometry of the cornea, while a viscoelastic boundary condition was applied at the limbal conjecture. This study and previous work Hwang et al. (2019); Lemke and Lucarelli (2012); Lockwood (1885); Banks et al. (2020); Comley and Fleck (2012) experimental and numerical findings were produced suggesting that the AFT is not the only form of support provided to the globe. The assumption of an axisymmetric geometry may be suitable for the cornea; however, Corvis corneal deformations show a very prominent occurrence of nasal rotation during retraction of the eye globe. Boszczyk et al. (2017) This nasal rotation drove the need to employ a three-dimensional geometrical set-up in this study, which implements irregularity and asymmetry of the orbital boundary. The orbital soft tissue has been the subject of several anatomical studies focusing on its structure. Sedlmayr (2002); Demer (2002) These studies report that most common eye movements involve sliding within the Tenon’s capsule of the OST. Indeed, Schoemaker et al. (2006) have attempted to estimate viscoelastic material properties by assessing the degree of deformation of AFT in eye globe rotation. In this study, a hyper-elastic material model was used to save computational time due to the Corvis pressure loading scenario’s simulation. This study differed from Shoemaker’s work in the loading conditions, where frontal loading was applied instead of the application of rotations onto the globe. The current study attempted to validate the hyper-elastic material model of OST using Corvis clinical corneal deformations, as suggested by Hwang et al. (2019).

In conclusion, this study utilised inverse finite element analysis with clinical measurements of the WEM under Corvis air pressure to estimate the OST’s stiffness and how this changes with age. The OST *E*_*t*_ has shown a weak correlation with age progression at the three different stress levels while showing significant differences between some age groups. With this information, numerical modelling of the eye globe, especially those simulating WEM, can now include models of the OST rather than introducing non-physiologic boundary conditions simulating its effect. Aboulatta et al. (2021); Jannesari et al. (2018) Nevertheless, despite consideration of the orbital geometry’s irregularity, numerical simulations did not accurately represent the nasal rotation aspect of clinical retraction of the eye globe. The authors of this study suggest that this rotation may be due to deeper orbital structures, such as EOMs, Lockwood’s ligament or other connective tissues. Lockwood (1885); Demer (2002) Thus, it is highly recommended to investigate the rotational response of the globe further, as this will have a considerable effect on future work regarding the simulation of impacts or exterior loading applied to the globe or even the orbital structure as a whole.

This study has consolidated previous findings, which stated the importance of the extraocular muscles in supporting the globe. Despite the age-related geometrical specification of the numerical model used in this study, the model needs improvement and requires the extraocular muscles to be added to it. This athors of this study believe that addition of the extraocular muscles will allow this numerical model to be an accurate representation of the ocular support system.

## Data Availability

The raw data supporting the conclusion of this article will be made available by the authors, without undue reservation.
